# The Steroidal Alkaloid Tomatidine and Tomatidine-Rich Tomato Leaf Extract Suppress the Human Gastric Cancer-Derived 85As2 Cells In Vitro and In Vivo via Modulation of Interferon-Stimulated Genes

**DOI:** 10.3390/nu14051023

**Published:** 2022-02-28

**Authors:** Junya Fujimaki, Neo Sayama, Shigenobu Shiotani, Takanori Suzuki, Miki Nonaka, Yasuhito Uezono, Mamoru Oyabu, Yasutomi Kamei, Haruo Nukaya, Keiji Wakabayashi, Akihito Morita, Tomoki Sato, Shinji Miura

**Affiliations:** 1 Laboratory of Nutritional Biochemistry, Graduate School of Nutritional and Environmental Sciences, University of Shizuoka, Shizuoka 422-8526, Japan; s20221@u-shizuoka-ken.ac.jp (J.F.); neo.sayama@primaham.co.jp (N.S.); momo.morita@nifty.com (A.M.); tsato1@u-shizuoka-ken.ac.jp (T.S.); 2 Food Research Institute, Tokai Bussan Co., Ltd., Tokyo 101-0032, Japan; shiotani@tokaibsn.co.jp (S.S.); tk-suzuki@tokaibsn.co.jp (T.S.); 3 Department of Pain Control Research, The Jikei University School of Medicine, Tokyo 105-8461, Japan; minonaka@jikei.ac.jp (M.N.); yuezono@jikei.ac.jp (Y.U.); 4 Laboratory of Molecular Nutrition, Graduate School of Life and Environmental Sciences, Kyoto Prefectural University, Kyoto 606-8522, Japan; oyabu0508@yahoo.co.jp (M.O.); kamei@kpu.ac.jp (Y.K.); 5 Food and Environment Research Center, Graduate School of Nutritional and Environmental Sciences, University of Shizuoka, Shizuoka 422-8526, Japan; nukaya@u-shizuoka-ken.ac.jp (H.N.); kwakabayashi@u-shizuoka-ken.ac.jp (K.W.)

**Keywords:** tomatidine, tomato leaves, gastric cancer, IFI27, type I interferon signaling pathway, type I interferon-stimulated genes

## Abstract

The steroidal alkaloid tomatidine is an aglycone of α-tomatine, which is abundant in tomato leaves and has several biological activities. Tomatidine has been reported to inhibit the growth of cultured cancer cells in vitro, but its anti-cancer activity in vivo and inhibitory effect against gastric cancer cells remain unknown. We investigated the efficacy of tomatidine using human gastric cancer-derived 85As2 cells and its tumor-bearing mouse model and evaluated the effect of tomatidine-rich tomato leaf extract (TRTLE) obtained from tomato leaves. In the tumor-bearing mouse model, tumor growth was significantly inhibited by feeding a diet containing tomatidine and TRTLE for 3 weeks. Tomatidine and TRTLE also inhibited the proliferation of cultured 85As2 cells. Microarray data of gene expression analysis in mouse tumors revealed that the expression levels of mRNAs belonging to the type I interferon signaling pathway were altered in the mice fed the diet containing tomatidine and TRTLE. Moreover, the knockdown of one of the type I interferon-stimulated genes (ISGs), interferon α-inducible protein 27 (*IFI27*), inhibited the proliferation of cultured 85As2 cells. This study demonstrates that tomatidine and TRTLE inhibit the tumor growth in vivo and the proliferation of human gastric cancer-derived 85As2 cells in vitro, which could be due to the downregulation of ISG expression.

## 1. Introduction

Gastric cancer had the fourth highest incidence rate and the third highest mortality rate among cancers worldwide in 2020 [1]. Gastric cancer risk is closely related to dietary habits, including an increased risk associated with salt intake [2,3] and preventive effects from fruit and vegetable consumption [4,5]. Accumulating evidence has shown that certain nutritional components, such as curcumin and resveratrol, have preventive effects against gastric cancer [6,7]. 

A steroidal glycoalkaloid α-tomatine is abundant in the flowers, leaves, calyxes, and unripe fruits of tomatoes (1.10, 0.98, 0.80, and 0.47 g/kg of fresh weight, respectively) [8]. Among these, leaves are unused resources that are discarded during tomato harvesting. α-Tomatine has various bioactivities, such as anti-cancer, anti-viral [9], and anti-inflammatory activities [10]. α-Tomatine also reduces the growth of several cultured cancer cell lines, such as LNCaP, VCaP, and PC-3 (human prostate cancer), K562 (human chronic myeloid leukemia), and HL60 (human acute promyelocytic leukemia) cells [11,12]. The anti-cancer activity of α-tomatine has also been shown in vivo, such as in the suppression of tumor formation in tumor-bearing mice using CT-26 (mouse colon cancer) cells via the intraperitoneal administration of α-tomatine (5 mg/kg body weight) [13]. The aglycone of α-tomatine, a steroid alkaloid tomatidine, has been reported to have similar bioactivity [14,15]. For example, incubation with 100 μM tomatidine for 48 h inhibited the growth of HT-29 (colon adenocarcinoma), HeLa (cervical carcinoma), and MCF-7 (breast adenocarcinoma) cells in vitro by 70, 60, and 80%, respectively [16]. Incubation with 30 μM tomatidine for 24 h also inhibited the growth of HBL-100 (breast cancer) cells by approximately 75% [17]. However, whether tomatidine inhibits tumor growth in vivo, similar to α-tomatine, has not yet been reported. Additionally, the effect of tomatidine on gastric cancer cells is unclear, as tomatidine inhibits the growth of the gastric cancer cell line KATO-III [18] but has no effect on another cancer cell line, AGS [19]. 

Therefore, we investigated whether tomatidine shows anti-cancer activity against human gastric carcinoma-derived 85As2 cells in vitro and its tumor in vivo and whether the same effect can be obtained with the tomatidine-rich tomato leaf extract (TRTLE) prepared from tomato leaves.

## 2. Materials and Methods

### 2.1. Preparation of TRTLE

Hot-air-dried tomato leaves (provided by Smart Agriculture Iwata Co., Ltd., Shizuoka, Japan) were added to a 15-fold volume (*v*/*w*) of water. After the pH was adjusted to approximately 3.5 with hydrochloric acid (Wako, Osaka, Japan), the sample was heated at 80 °C for 30 min, and diatomaceous earth filtration was performed to obtain the extract. Trisodium citrate dihydrate (Wako) was added to 4% (*w*/*w*) concentration of the extract, and the pH was adjusted to 8.5 with sodium hydroxide (Wako). After centrifugation at 4000× *g* for 10 min at 25 °C, the precipitate was resuspended in water, and the solution was centrifuged again at 4000× *g* for 10 min at 25 °C. To hydrolyze α-tomatine in the extract, the water-washed precipitate was dissolved in 1.3 mol/L of hydrochloric acid, and the solution was heated at 80 °C for 2 h. After hydrolysis, the pH was adjusted to 8.5, and the precipitate containing hydrolyzed α-tomatine was obtained after centrifugation at 4000× *g* for 10 min at 25 °C and washing with water as described above. TRTLE was obtained by freeze-drying the precipitate. The tomatidine and α-tomatine contents in TRTLE were determined by high-performance liquid chromatography (HPLC) analysis using SPD-10A (detector) and SCL-10 (pump) (Shimadzu, Kyoto, Japan) as described by Taveira et al. [20]. Samples were dissolved in methanol, diluted with the same solvent, and filtered through a membrane filter (0.45 µm) (Pall corporation, Port Washington, New York, NY, USA). Ten microliters of the sample were injected into the HPLC system, and quantification was carried out using a calibration curve prepared with the tomatidine standard. The HPLC conditions are listed in Table 1. A-Tomatine and tomatidine hydrochloride were purchased from the Tokyo Chemical Industry (Tokyo, Japan) and Sigma-Aldrich Japan (Tokyo, Japan), respectively. 

### 2.2. Cell Culture

Human gastric cancer-derived 85As2 cells were established from the human gastric cancer (MKN-45) cell line as described previously [21] and cultured in Roswell Park Memorial Institute-1640 (Wako) medium with 10% fetal bovine serum (FBS; Sigma-Aldrich) and 1% penicillin-streptomycin solution (stabilized) (Nacalai Tesque, Kyoto, Japan). The cells were cultured in a humidified atmosphere at 5% CO_2_ and 37 °C, and the medium was replaced every two days. 85As2 cells were seeded at 1.5 × 10^4^ or 3.0 × 10^3^ cells/well in a 24- or 96-well plate (As One, Osaka, Japan), respectively. One day after seeding, the cells were treated with the vehicle, 6.5 μg/mL tomatidine (hydrochloride) (Cayman chemical, Ann Arbor, MI, USA), or 10 μg/mL TRTLE (equivalent to 6.5 μg/mL tomatidine) for 24 h, 48 h, or 72 h. Dimethyl sulfoxide (DMSO; Wako) was used to dissolve the compounds, and the final concentration of DMSO in the cells was 0.1%.

### 2.3. Cancer Model Induced by Implantation of 85As2 Cells

Eight-week-old male BALB/c-AJcl-nu/nu mice were purchased from Clea Japan, Inc. (Tokyo, Japan) and acclimatized for a week. The mice were provided free access to water and a normal chow diet (MF; Clea, Japan) and maintained under a 12 h light/dark cycle at a constant temperature of 22 °C. To create a syngeneic mouse model, 85As2 cells were harvested from subconfluent cultures and resuspended in phosphate-buffered saline (PBS). Mice anesthetized by inhalation of 1–2.5% isoflurane were subcutaneously inoculated with 1 × 10^6^ cells at each site in the left and right flanks. After cell transplantation, the mice were fed a control diet (Control group), a diet containing 0.05% (*w*/*w*) tomatidine (Tomatidine group), or 0.077% (*w*/*w*) TRTLE (equivalent to 0.05% (*w*/*w*) tomatidine) (TRTLE group) for 3 weeks. 

All components of the diet are listed in Table 2. After mixing all the powders together, oil and an appropriate amount of water were added while stirring to solidify the contents. The diets were stored at −30 °C.

All animal experiments were approved by the Institutional Animal Care and Use Committee of the University of Shizuoka (protocol codes 195240 and 215299; date of approval: 13 June 2019, and 25 March 2021, respectively).

### 2.4. Microarray Analysis

RNA samples were extracted from tumors from the 85As2 syngeneic mouse model using the RNeasy Fibrous Tissue Mini Kit (Qiagen, Venlo, The Netherlands). The obtained RNA samples were pooled for each group (Control group, *n* = 4; Tomatidine group, *n* = 5; TRTLE group, *n* = 5) and used for microarray analysis. Microarray analysis was performed by the DNA Chip Research Inc. (Tokyo, Japan) using SurePrint G3 Human GE microarray 8 × 60 K Ver3.0 chips.

From the microarray data, we identified 90 genes in the Tomatidine group and 115 genes in the TRTLE group with an absolute expression level of at least 500 in the Control group and a fold change of at least 1.3 both in the Tomatidine and TRTLE groups as compared to the Control group. To find a common set of differentially expressed gene clusters, these genes were uploaded to the Search Tool for the Retrieval of Interacting Genes/Proteins (STRING) database (http://string-db.org/, 28 January 2022), and gene ontology (GO) analysis was performed.

### 2.5. Quantitative Reverse Transcription-Polymerase Chain Reaction (qRT-PCR) Analysis

Total RNA was extracted from the tumor-derived 85As2 syngeneic mouse model or cultured 85As2 cells after 72 h of incubation using an RNA Extraction kit (Takara Bio Inc., Shiga, Japan), according to the manufacturer’s protocol. The obtained RNA was subjected to gDNA removal and reverse transcription using the PrimerScript^TM^ RT Reagent Kit (Takara Bio Inc.) to produce cDNA. PCR reactions were performed with TB Green^®^ Premix Ex Taq™ II (TliRNaseH Plus) and Bulk (Takara Bio Inc.) using the Thermal Cycler Dice^®^ Real Time System (Takara Bio Inc.). cDNA (7.5 ng) and 10 nmol gene-specific primers were used for amplification in 12.5 μL qRT-PCR reaction mixture. The qPCR reactions were carried out at 95 °C for 30 s, followed by 40 cycles at 95 °C for 5 s and 60 °C for 30 s, and then terminated with a dissociation step for 15 s at 92 °C, 30 s at 60 °C, and 15 s at 95 °C. Target gene expression was calculated using the Ct value (second derivative maximum) methods normalized to β-actin or glyceraldehyde 3-phosphate dehydrogenase (GAPDH) expression. The primer sequences used for qRT-PCR are listed in Table 3.

### 2.6. Cell Proliferation and Cytotoxicity Assays

Cell proliferation assays were performed using a cell counting kit-8 (CCK-8) (Dojindo, Tokyo, Japan), and cytotoxicity was assessed using the Cytotoxicity lactate dehydrogenase (LDH) Assay Kit-WST (Dojindo). The 85As2 cells (3 × 10^3^ cells per well) were seeded into a 96-well plate. After incubation for the time shown in the figure, the cell proliferation and cytotoxicity were evaluated according to the manufacturer’s protocol. Cytotoxicity (%) = [absorbance] (sample—negative control)/(positive control—negative control) × 100. The positive control was cells treated with the lysis buffer, and the negative control was untreated cells.

### 2.7. Small Interfering RNA (siRNA) Transfection

Transfection of siRNA into 85As2 cells was performed according to a standard protocol. The cells were transfected with 10 nM siRNA using Lipofectamine RNAiMAX (Invitrogen, Tokyo, Japan) the day after seeding. The cells were collected after 72 h of incubation and analyzed using qRT-PCR to determine the knockdown efficiency. siRNA oligonucleotides were purchased from Japan Bio Service (JBioS, Saitama, Japan). Two independent siRNAs for each gene were tested, and the oligo sequences of all siRNAs are listed in Table 4. The lowercase “d” at the 3’ terminus indicates an overhang in the DNA sequence.

### 2.8. Statistical Analysis

All data are expressed as the mean ± standard error of the mean (SEM). Statistical analyses were performed using JMP software (JMP 5.1.2; SAS, Cary, NC, USA). One-way analysis of variance followed by Dunnett’s test was performed for comparison with the control sample.

## 3. Results

### 3.1. HPLC Analysis of TRTLE

**Figure 1:**
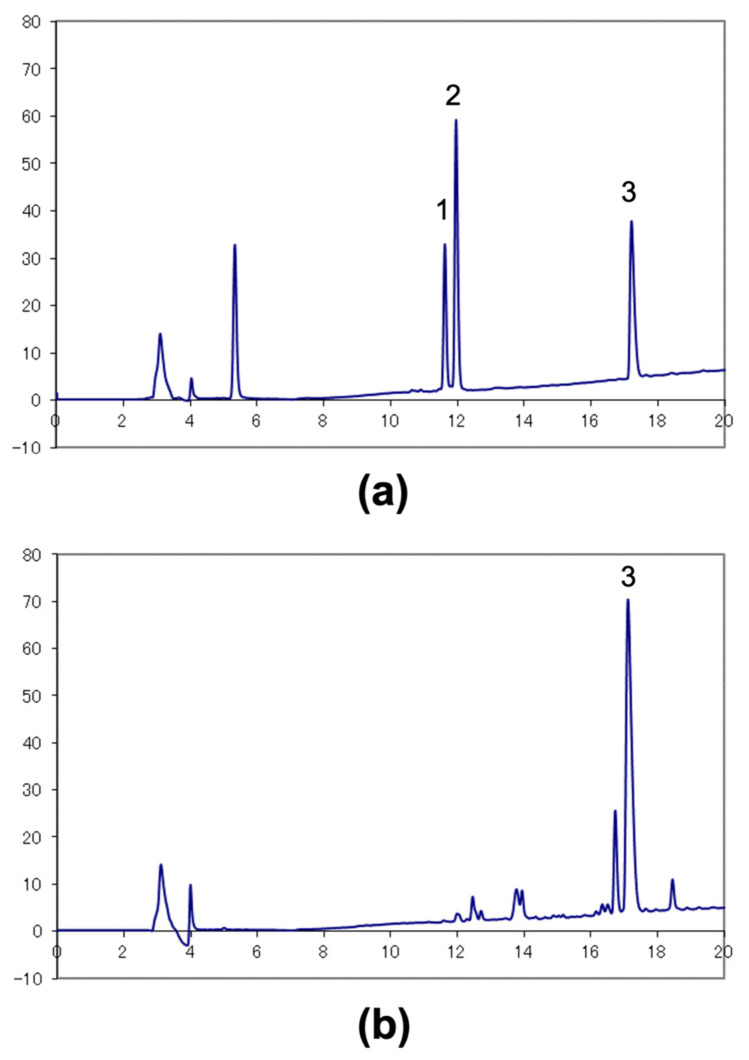
High-performance liquid chromatography (HPLC) chromatograms of α-tomatine and tomatidine standards (**a**) and the tomatidine-rich tomato leaf extract (TRTLE) (**b**). Peaks from (1) dehydrotomatine, (2) α-tomatine, and (3) tomatidine.

Commercial α-tomatine and tomatidine standards were simultaneously analyzed. The HPLC chromatogram showed peaks (1) and (2), at retention times of 11.6 and 12.0 min, respectively (Figure 1a), similar to that reported by Taveira et al. [20]. They reported that the former peak was dehydrotomatine, and the latter was α-tomatine. Peak (3) at 17.2 min was tomatidine. Peaks corresponding to dehydrotomatine and α-tomatine were not detected in the HPLC chromatogram of TRTLE (Figure 1b), suggesting that these tomatines are almost completely aglyconated by acid hydrolysis. The concentration of tomatidine in TRTLE was 65% (*w*/*w*), and α-tomatine was not detected (Figure 1b). Taveira et al. reported that two peaks were detected in the HPLC chromatogram of the tomatidine standard, eluted in the order of: tomatidenol, aglycone of dehydrotomatine, and tomatidine [20]. Although for the HPLC chromatogram of the commercial tomatidine standard used in this study, only one peak (3) was detected (Figure 1a), two peaks were detected in the HPLC chromatogram of the acid-hydrolyzed commercial α-tomatine standard (retention time 16.8 min and 17.2 min, results not shown). These results suggest that the peak detected at a retention time of 16.8 min in TRTLE was most likely to be tomatidenol (Figure 1b). 

### 3.2. Tomatidine and TRTLE Inhibit Tumor Growth in a Syngeneic Mouse Model

**Figure 2:**
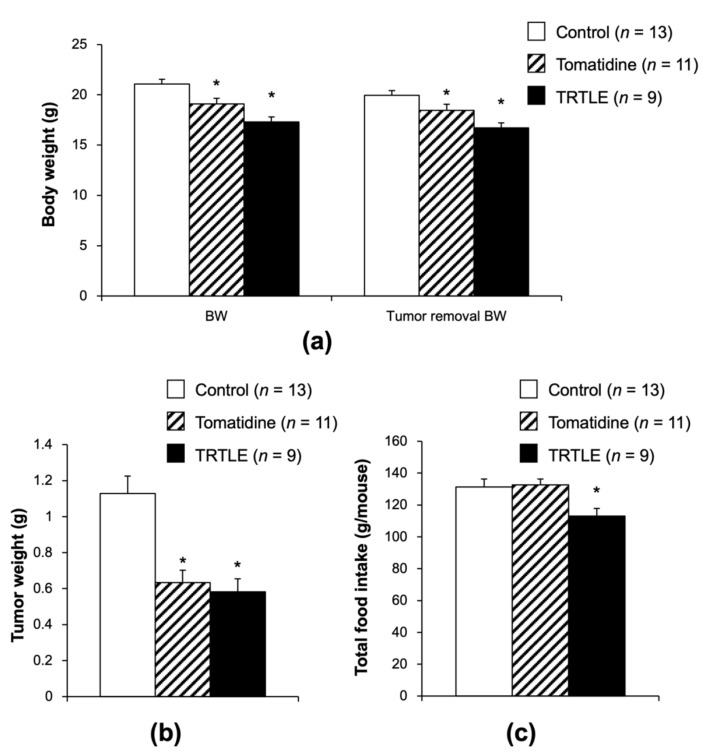
Effect of tomatidine and TRTLE on the tumor growth of an 85As2 syngeneic mouse model. Eight-week-old male BALB/c-AJcl-nu/nu mice were subcutaneously injected with 85As2 cells derived from human gastric cancer tissues. The mice were fed control diets (Control) or diets containing tomatidine (Tomatidine) or TRTLE (TRTLE) for 3 weeks. (**a**) Body weight (BW), BW after tumor removal (Tumor removal BW), and (**b**) tumor weight after 3-week experimental period are shown. (**c**) Total food intake during the 3 weeks of the experimental period is also shown. *n* = 9–13, means ± standard error (SE). * *p* < 0.05 vs. Control.

We investigated the effect of tomatidine and TRTLE on tumor formation using a cancer model involving the implantation of 85As2 cells. Body weight and tumor removal body weight were decreased in the Tomatidine and TRTLE groups (Figure 2a). Tumor weight was remarkably reduced to 56.1% and 51.6% in the Tomatidine and TRTLE groups, respectively (Figure 2b). Total food intake for 3 weeks was reduced only in the TRTLE group. These data suggest that the administration of tomatidine and TRTLE inhibited tumor growth in vivo. 

### 3.3. Tomatidine Suppresses the Expression Levels of Type I Interferon-Stimulated Genes

**Figure 3:**
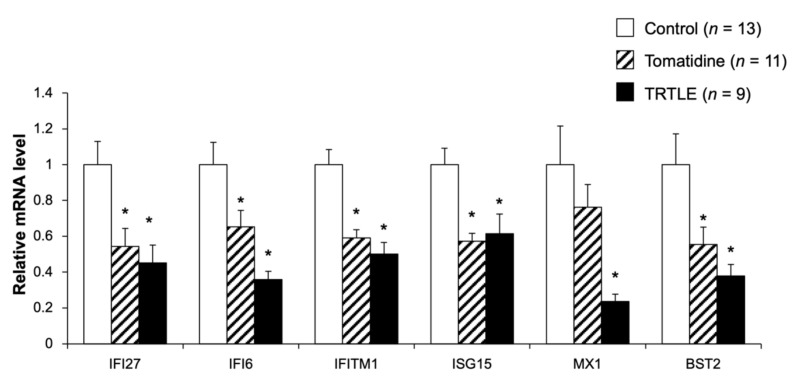
Expression levels of the type I interferon-stimulated genes in tumors derived from the cancer mouse model induced by the implantation of 85As2 cells. To confirm the effects of tomatidine and TRTLE, gene expression levels in tumors from the cancer mouse model fed control diet (Control) or diets containing tomatidine (Tomatidine) or TRTLE for 3 weeks were measured using quantitative reverse transcription-polymerase chain reaction (qRT-PCR). *n* = 9–13, means ± SE. * *p* < 0.05 vs. Control.

To investigate the mechanism of tomatidine- and TRTLE-induced suppression of tumor growth, microarray analysis was performed on excised tumor tissues, and GO analysis of the obtained data showed that the expression of mRNAs belonging to the type I interferon signaling pathway was altered in the mice fed the diet containing tomatidine or TRTLE (Table 5 and Table 6). The results of Tomatidine versus Control indicated only four descriptions (Table 5). On the other hand, TRTLE vs. Control showed 48 descriptions; the top 10 in the order of decreasing *p*-value are shown (Table 6). Among these were six genes, namely, interferon-induced protein 27 (*IFI27*), interferon-induced protein 6 (*IFI6*), interferon-induced transmembrane protein 1 (*IFITM1*), interferon-stimulated gene 15 (*ISG15*), Mx dynamin-like GTPases (*MX1*), and bone marrow stromal cell antigen 2 (*BST2*), that were commonly altered in both the Tomatidine and TRTLE groups. When the expression of these genes was measured using qRT-PCR, all genes except *MX1* showed significant changes in both groups (Figure 3). These genes are known as type I interferon-stimulated genes (ISGs), and ISGs may be involved in the suppression of tumor growth by tomatidine.

### 3.4. Tomatidine Inhibits the Proliferation of 85As2 Cells without Cytotoxicity

**Figure 4:**
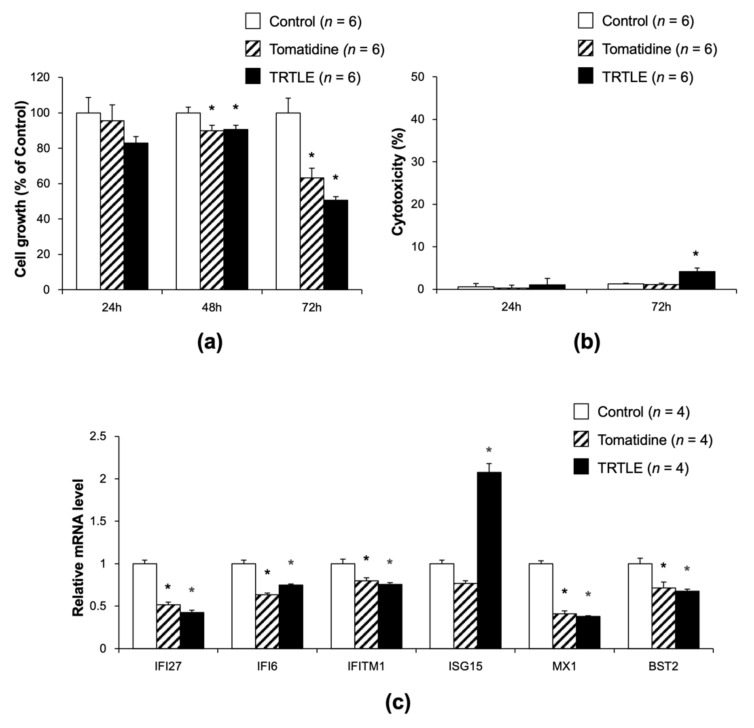
Effects of tomatidine and TRTLE on the proliferation of 85As2 gastric cancer cells. After incubation of 85As2 cells for 24 h, 48 h or 72 h without (Control) or with tomatidine (Tomatidine) or TRTLE, their (**a**) cell growth and (**b**) cytotoxicity were measured. (**c**) mRNA levels of type I interferon-stimulated genes (ISGs) were measured in the 85As2 cells treated with tomatidine or TRTLE for 72 h. *n* = 4–6, means ± SE. * *p* < 0.05 vs. Control.

Next, we examined whether tomatidine directly affected the proliferation of 85As2 cells and/or was cytotoxic in these cells. Although 24 h of incubation with tomatidine or TRTLE did not show any significant effect on cell proliferation, tomatidine and TRTLE inhibited cell growth after 48 and 72 h incubation. After 72 h, tomatidine and TRTLE inhibited cell growth to 63.3% and 50.7%, respectively (Figure 4a). Cytotoxicity was not observed after 72 h of incubation with tomatidine (Figure 4b). TRTLE showed cytotoxicity; however, the 50.7% suppression of cell growth after 72 h incubation with TRTLE could not be explained by only a 4.1% cytotoxicity. We also measured the expression levels of ISGs in 85As2 cells cultured in a medium containing tomatidine and TRTLE for 72 h. The expression levels of these genes, except *ISG15*, were significantly decreased in the cells cultured with tomatidine and TRTLE (Figure 4c). These results indicate that tomatidine and TRTLE inhibit tumor growth in vivo and proliferation of 85As2 gastric cancer cells in vitro. Additionally, the expression levels of ISGs, except *ISG15*, were also decreased both in vivo and in vitro. 

### 3.5. Knocking Down of IFI27 Inhibits the Proliferation of 85As2 Cells

**Figure 5:**
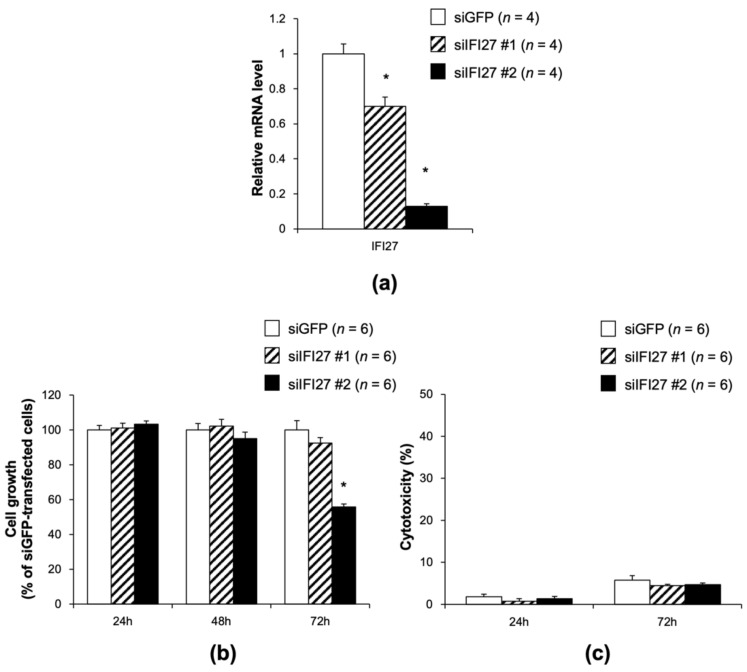
Effect of the knockdown of interferon α-inducible protein 27 (*IFI27*) on the proliferation of 85As2 gastric cancer cells. (**a**) Seventy-two hours after transfection with siIFI27, IFI27 mRNA levels in 85As2 cells were measured. After 24 h, 48 h or 72 h of transfection with siIFI27, (**b**) cell growth and (**c**) cytotoxicity were measured. siGFP was transfected as a control. *n* = 4–6, means ± SE. * *p* < 0.05 vs. Control.

To investigate whether tomatidine- and TRTLE-induced suppression of ISG expression was required for the inhibition of 85As2 cell proliferation, two types of siIFI27 (#1, #2) were prepared and transfected into 85As2 cultured cells to generate IFI27 knockdown cells. Both siIFI27s (#1, #2) showed a significant decrease in *IFI27* gene expression after 72 h of incubation (Figure 5a), with the knockdown efficiency higher in cells treated with siIFI27 #2 than in those treated with #1. Knockdown of *IFI27* after 72 h incubation with siIFI27 #2 inhibited cell growth to 55.7% (Figure 5b). Knockdown of *IFI27* showed no cytotoxicity in 85As2 cells (Figure 5c), suggesting that cytotoxicity was not involved in the decrease in cell growth by knocking down *IFI27*. These results were at a similar level to that of the changes caused by tomatidine and TRTLE. Therefore, tomatidine and TRTLE may contribute to the inhibition of cancer cell growth and tumor formation through the downregulation of expression of ISGs, such as *IFI27*.

## 4. Discussion

In this study, we attempted to elucidate the anti-cancer effects of tomatidine and TRTLE and their underlying mechanisms. We have shown that tomatidine and TRTLE have anti-cancer effects on human gastric cancer-derived 85As2 cells in vivo and in vitro, using a syngeneic mouse model and growth assays with cultured cells, respectively. Furthermore, microarray analysis suggested that tomatidine and TRTLE could regulate ISGs.

The results of the present study are the first to demonstrate the anti-cancer activity of tomatidine in vivo (Figure 2b). Similar results were obtained with TRTLE. Previously, it was reported that the administration of tomatidine (subcutaneously, 25 mg/kg/day for 10 days) did not show anti-cancer effects in vivo [22]. In this study, the administration of tomatidine was initiated when a maximum tumor diameter of 5 mm was observed. However, in our present study, approximately 160 mg/kg/day of tomatidine was administered orally for 3 weeks, and administration was started on the day of 85As2 cell transplantation. The difference in results might be attributable to the starting time, dose, method, schedule of administration, or the cell line used. 

Administration of tomatidine and TRTLE reduced tumor weight but also resulted in a significant decrease in body weight. Although the decrease in body weight observed in the TRTLE group might be caused by reduced total food intake, a decrease in body weight in the tomatidine group was observed without a reduction in total food intake. This may be related to the effect of tomatidine on lipid metabolism. It has been reported that tomatidine suppresses high-fat diet-induced increases in body weight and fat accumulation in white adipose tissue [23]. Additionally, tomatidine suppresses lipid accumulation in HepG2 hepatocytes [24] and reduces hepatic lipid accumulation in mice fed a high-fat diet, by suppressing the expression of fatty acid synthases and transcription factors involved in lipogenesis [23]. One might hypothesize that tomatidine-induced changes in lipid metabolism may cause a decrease in body weight.

The contribution of cytotoxicity to the effect of tomatidine seemed to be low, compared to that of the anti-cancer effect of α-tomatine. When C6 rat glioma cells were treated for 4 h at 10 and 20 μM of α-tomatine, 40 and 80% cytotoxicity based on LDH activity were observed, respectively [25]. The cytotoxicity of α-tomatine has been suggested to be due to a membrane-disrupting effect, as it can bind to cholesterol [26]. On the other hand, in the present study, an increase in LDH activity was not observed when 85As2 cells were treated with 6.5 μg/mL (14.4 μM) of tomatidine for 72 h (Figure 4b). Therefore, we suggest that tomatidine may exert its anti-cancer effects through a mechanism other than α-tomatine-induced cytotoxicity. 

Microarray results showed that tomatidine affected ISGs. Of the six genes identified, *IFI27*, *IFI6*, *IFITM1*, *ISG15*, and *BST2* have been reported to be associated with cancer cell proliferation and tumor growth. Overexpression of *IFI27* promotes cancer cell proliferation and tumor growth [27], while its knockdown exhibits the opposite effect [28,29]. *IFI6* depletion inhibits esophageal squamous cell carcinoma progression [30]. *IFITM1* deficiency suppresses the proliferation, metastasis, and invasion of lung cancer cells [31]. Upregulation of *ISG15* expression promotes colon cancer proliferation and metastasis, and when *ISG15* is knocked down, there is an opposite effect [32]. Knocking down *BST2* suppresses gastric cancer cell proliferation and promotes apoptosis [33]. Therefore, it is suggested that the downregulation of ISG expression contributes to tomatidine- and TRTLE-induced suppression of 85As2 cell proliferation and tumor growth. In the present study, we focused on *IFI27*. *IFI27* is highly expressed in several cancers, such as ovarian cancer [34,35], hepatocellular carcinoma [36], and breast cancer [37]. In fact, *IFI27* is a potential prognostic marker and therapeutic target for cancer and tumor growth [38,39,40]. High expression of *IFI27* inhibits apoptosis [27,28], and its deficiency promotes apoptosis [39]. *IFI27* is also involved in the transition from the S phase to the G2 phase in the cell cycle [29,35] and affects the expression of VEGF-A, which is required for the promotion of angiogenesis [29]. Furthermore, *IFI27* was found to be closely associated with the clinical progression of pancreatic adenocarcinoma (PAAD), tumor glycolysis, and M2 macrophage infiltration [40]. Downregulation of *IFI27* expression might be partially involved in tomatidine- and TRTLE-induced inhibition of 85As2 cancer cell proliferation and tumor growth. Additionally, the expression of ISGs is upregulated in tumors and are positively correlated with resistance to radiotherapy [41,42]. The immune-checkpoint protein PD-L1 has been reported to protect cancer cells by suppressing DNA damage through the inhibition of the acute response to IFN-1 and the maintenance of ISG expression [43]. Thus, we hypothesize that tomatidine could reduce the resistance of cancer cells to treatment by downregulating ISG expression and may be useful as an adjuvant therapy for radiotherapy. As the relationship between gastric cancer and *IFI27* and other ISGs is still unclear, further studies are required.

## 5. Conclusions

Tomatidine and TRTLE inhibited the tumor formation and growth of cultured 85As2 cells derived from human gastric cancer tissues. This is the first demonstration of the anti-cancer activity of tomatidine in vivo. Furthermore, the results suggest that the anti-cancer activities of tomatidine and TRTLE are mediated by the downregulation of expression of ISGs, such as *IFI27*.

## 6. Patents

The preparation method for TRTLE used in this study is the subject of a patent application (JP 2018-184394A) by Tokai Bussan Co., Ltd. 

## Figures and Tables

**Table 1:** High-performance liquid chromatography (HPLC) conditions for tomatidine and α-tomatine concentration measurements.

HPLC	
Column	CAPCELL PAK C18-ACR column 4.6 × 250 mm (OSAKA SODA, Osaka, Japan)
Guard column	CAPCELL PAK C18-ACR guard column 4.0 × 10 mm (OSAKA SODA)
Wavelength	205 nm
Mobile phase A	25 mM Triethylammonium phosphate
Mobile phase B	Acetonitrile
Gradient	B% = 20–45–55–57–20 (0–12, 12–17, 17–20, 20–21, 21 min)
Column temperature	25 °C
Flow rate	0.8 mL/min

HPLC: High-performance liquid chromatography.

**Table 2:** Composition of the diet containing tomatidine or tomatidine-rich tomato leaf extract (TRTLE).

Ingredients	Control	Tomatidine	TRTLE
	g/100 g (Except Water)		
Tomatidine (hydrochloride)	-	0.05	-
Tomatidine-rich tomato leaf extract (TRTLE)	-	-	0.077
L(-)-Cystine (Wako)	0.30	0.30	0.30
AIN93 Vitamin mix (Without choline bitartrate) (Oriental Yeast, Tokyo, Japan)	1.00	1.00	1.00
AIN93 Mineral mix (Oriental Yeast)	3.50	3.50	3.50
Cellulose (Oriental Yeast)	5.00	5.00	5.00
Casein (Oriental Yeast)	20.00	20.00	20.00
α-Starch (Oriental Yeast)	66.20	66.20	66.20
Safflower oil (Benihana Olein Ichiban Shibori) (Benibana foods, Tokyo, Japan)	4.00	4.00	4.00

**Table 3:** Sequences of primers used for quantitative reverse transcription-polymerase chain reaction (qRT-PCR).

Gene	Forward (5′–3′)	Reverse (5′–3′)
β-actin	TGGCACCCAGCACAATGA	CTAAGTCATAGTCCGCCTAGAAGCA
*GAPDH*	TGGACCTGACCTGCCGTCTAG	GTGGGTGTCGCTGTTGAAGTC
*IFI27*	TGCTCTCACCTCATCAGCAGT	CACAACTCCTCCAATCACAACT
*IFI6*	GATGAGCTGGTCTGCGATCC	TCGAGATACTTGTGGGTGGC
*IFITM1*	TCGCCTACTCCGTGAAGTCTA	TGTCACAGAGCCGAATACCAG
*ISG15*	TGTCCCTGAGCAGCTCCATG	TGTCCTGCAGCGCCACACC
*MX1*	GCCAGGACCAGGTATACAG	GCCTGCGTCAGCCGTGC
*BST2*	GAGCTTGAGGGAGAGATCACTAC	ATTCTCACGCTTAAGACCTGGTT

*GAPDH*, glyceraldehyde 3-phosphate dehydrogenase; *IFI27*, interferon-induced protein 27; *IFI6*, interferon-induced protein 6; *IFITM1*, interferon-induced transmembrane protein 1; *ISG15*, interferon-stimulated gene 15; *MX1*, Mx dynamin-like GTPases; *BST2*, bone marrow stromal cell antigen 2.

**Table 4:** Sequences of small interfering RNAs (siRNAs).

siRNA	Sense (5′–3′)	Antisense (5′–3′)
GFP	GCAGCACGACUUCUUCAAGdTdT	CUUGAAGAAGUCGUGCUGCdTdT
IFI27 #1	GUGAAAUAUACCAAAUUCUdTdT	AGAAUUUGGUAUAUUUCACdCdC
IFI27 #2	GAAAUAAAGAUGAAUUGUUdTdT	AACAAUUCAUCUUUAUUUCdTdT

GFP, green fluorescent protein; IFI27, interferon-induced protein 27.

**Table 5:** Results of gene ontology (GO) analysis in biological processes (Tomatidine vs. Control).

Biological Process (Gene Ontology)			
GO-Term	Description	Count in Network	*p*-Value
GO:0060337	Type I interferon signaling pathway	6 of 67	0.0062
GO:0045069	Regulation of viral genome replication	6 of 99	0.0114
GO:1903900	Regulation of viral life cycle	7 of 153	0.0114
GO:0045071	Negative regulation of viral genome replication	5 of 61	0.0154

**Table 6:** Top 10 results of GO analysis in biological processes (TRTLE vs. Control).

Biological Process (Gene Ontology)			
GO-Term	Description	Count in Network	*p*-Value
GO:0040029	Regulation of gene expression, epigenetic	12 of 202	4.63 × 10^−6^
GO:0060968	Regulation of gene silencing	10 of 137	1.12 × 10^−5^
GO:0045814	Negative regulation of gene expression, epigenetic	9 of 103	1.28 × 10^−5^
GO:0097549	Chromatin organization involved in negative regulation of transcription	9 of 108	1.42 × 10^−5^
GO:0006334	Nucleosome assembly	9 of 135	5.89 × 10^−5^
GO:0045653	Negative regulation of megakaryocyte differentiation	5 of 18	0.00012
GO:0060337	Type I interferon signaling pathway	7 of 67	0.00012
GO:0006323	DNA packaging	10 of 215	0.00012
GO:0051253	Negative regulation of RNA metabolic process	23 of 1422	0.00012
GO:0051172	Negative regulation of nitrogen compound metabolic process	31 of 2429	0.00012

## Data Availability

The data obtained in this study will be shared upon reasonable request to the corresponding author.
